# Effect of NaCl concentration on stability of a polymer–Ag nanocomposite based Pickering emulsion: validation *via* rheological analysis with varying temperature

**DOI:** 10.1039/d0ra03199b

**Published:** 2020-06-04

**Authors:** Ramesh Narukulla, Umaprasana Ojha, Tushar Sharma

**Affiliations:** Department of Chemistry, Rajiv Gandhi Institute of Petroleum Technology Jais Bahadurpur, Mukhetia More, Harbanshganj Amethi Uttar Pradesh-229304 India; Department of Petroleum Engineering, Rajiv Gandhi Institute of Petroleum Technology Jais Bahadurpur, Mukhetia More, Harbanshganj Amethi Uttar Pradesh-229304 India tsharma@rgipt.ac.in +91-7080044156

## Abstract

The utility of a Pickering emulsion (PEm) under saline conditions is strongly dependent on the stability of the emulsion in the presence of different salt concentrations. In this study, we have evaluated the effect of NaCl and temperature on the stability of a polyacryloyl hydrazide (PAHz)–Ag nanocomposite (NC) based PEm utilizing ocular observation, an optical microscope with a thermal stage, TGA, DLS, electrical conductivity, and rheological studies at different temperatures. The creaming stability of PEm in the presence of salt concentrations in the range of 0.5 to 5.0 wt% was evaluated by adding different amounts of PAHz–Ag NC stabilizer. The effect of NaCl on emulsion stability is strongly dependent on the aggregation behaviour of the nanocomposites, showing signs of aggregation at low NaCl concentration (<3 wt%) and re-dispersion at high NaCl concentration (>3 wt%). As per the microscopy analysis, 25 wt% of PAHz–Ag NC was sufficient to stabilize the PEm for a period of up to 7 days in the presence of salt concentrations up to 3 wt% in the aqueous layer at 95 °C. Thus, the balance of ionic strength provides an important insight into the nature of nanocomposite interactions in emulsion systems and a possible mechanism for designing emulsion properties *via* salt inclusion.

## Abstract 2

Schematic for the impact of NaCl on droplet stabilization in Pickering emulsions.

## Graphical abstract

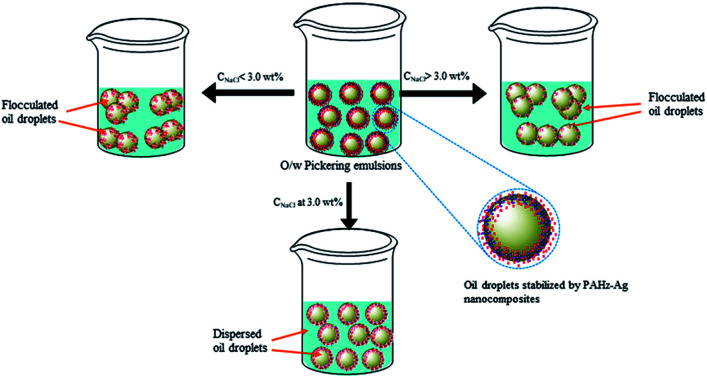

## 1. Introduction

Pickering emulsions (PEm) have vital roles in various industries like pharmaceuticals, food, cosmetics, drug delivery, automobiles, and oil recovery.^1^ Solid particles like silica,^2^ whey protein nanoparticles,^3^ silver nanoparticles,^4^ quinoa protein (QPI) nanoparticles,^5^ cellulose nanofibers,^6^*etc.* are generally used to prepare Pickering emulsions. Unlike conventional surfactants, the oil-water interface can be adsorbed by nanoparticles more efficiently due to their small size and greater surface area.^7,8^ As a result, PEm offer a wide range of advantages such as enhanced thermal stability, restricted coalescence of emulsion droplets, and delayed creaming. Therefore, PEm can be stored for longer duration at various conditions as demonstrated by Narukulla *et al.*^4^ who prepared PEm of polymer polyacryloyl hydrazide (PAHz) and Ag NPs; emulsion stabilized by high PAHz concentration (25 wt%) and Ag NPs (∼10 nm) was stable over a storage period of 30 days. Unfortunately, electrolytic strength of aqueous phase leads to disruption of emulsion droplets resulting oil droplets coalesce and separate from the emulsion.^9^ The trait that makes Pickering emulsion stable is thicker interfacial layer of NPs which may resist creaming and droplet coalescence against addition of salt.^10^ Thus, compared to normal emulsions, salt treated PEm should be more attractive to study as a result of their variable behavior in presence of salinity.^11–13^ Some studies, reported the effect of salt (NaCl, LaCl_3_, tetraethyl ammonium bromide, CaCl_2_, and MaCl_2_) on PEm, were based on hydrophilic fumed silica particles,^14^ clay particles,^15^ laponite particles,^16^ positively charges plate-like layered double hydroxides (LDHs),^17^ grapheme oxide,^18^ chitosan-tripolyphosphate (CS-TPP),^19^ and zein/gum arabic nanoparticles (ZGPs).^20^ However, size distribution of droplet,^21,22^ type of emulsion,^23^ interfacial tension values,^24^ interfacial rheology,^24,25^ droplet coalescence,^26^ emulsion properties influenced by temperature,^22^ and oil recovery^27^ have been mostly the focus of discussion, and limited information, for the influence of varying salinity on stability and flow properties of PEm stabilized by PAHz–Ag NPs, is available on which we focus here.

Rheology is an important tool to estimate the effect of electrolyte on emulsion behavior such as stability, deformation of droplets, flow behavior, and viscoelastic properties.^28,29^ Generally, droplet deformation depends on droplet size, thickness of interfacial layer, and viscosity difference in emulsion phases.^30^ Similarly, flow behavior of emulsion depends on droplet size, aqueous phase volume fraction, adsorbed particles concentration, and interfacial layer.^31^ Addition of salt may change the flow properties by improving the thickness of interfacial NP layer^32^ or by removing the layer,^33^ and these changes can be best understood by rheological analysis. Viscosity is one of the critical flow properties of emulsions and its variation with increasing salt concentration for SPI (soy protein isolate) stabilized emulsions is studied.^34^ With lower NaCl concentration (100 mM), the viscosity of emulsion was found higher than the one without NaCl. At higher NaCl concentration (400 mM), the viscosity decreased and its value became lower than the value associated with emulsion without NaCl.^34^ Apart from viscosity, it is also important to study viscoelastic properties of PEm which can be envisaged by frequency-sweep analysis. Frequency-sweep analysis gives information on viscous (loss modulus = *G*′′) and elastic nature (storage modulus = *G*′) of the emulsion. In emulsions, the elastic strength of interfacial NP layer usually modifies by NaCl concentration resulting it leads to significant change in viscoelastic properties (*G*′ and *G*′′) of emulsion.^35^ Horozov *et al.*^10^ studied the effect of NaCl on the fumed silica particle stabilized silicone o/w emulsions and found that *G*′ rapidly increased at higher ionic strength (>2 mM). *G*′ of emulsion was below 100 (Pa) at lower ionic strength (<1 mM) and it increased to 200 Pa at ionic strength (>2 mM). The study demonstrated that interaction between particle and droplets were was favorable at higher ionic strength which as a result, increased the three dimensional network (gel) while lower ionic strength made emulsion unstable by showing significant creaming. In another study, NaCl (3.5 wt%) addition to decane-in-water PEm, stabilized by fumed hydrophilic silica particles (Aerosil R7200), showed significant improvements in elasticity of emulsion resulting *G*′ became 10 times higher than *G*′′.^36^ The effect of varying NaCl concentration (0.1–10 wt%) on stability of PEm, stabilized by NPs (SiO_2_ and clay) and surfactant (sodium dodecyl sulfate, SDS) in base fluid of polymer, was studied for oilfield applications^37^ where 1 wt% NaCl was found favorable for the synthesis of stable emulsion. In addition, these emulsions showed viscoelastic behavior at lower frequency (<9 rad s^−1^) and viscous behavior (*G*′′) at higher frequency (>9 rad s^−1^), which clearly demonstrates the importance of evaluating the influence of electrolytes on rheological behavior of PEm. Thus, this study reports the influence of varying salinity on stability and rheological properties of PEm, stabilized by polyacryloyl hydrazide (PAHz)–Ag NC, for industrial applications such as food packaging and pharmaceuticals, which has not been reported in literature as far as aware.

In our previous study, we have reported a stable dried and re-dispersible o/w PEm (∼98% oil content) stabilized by PAHz–Ag NC.^4^ Where we studied varying Ag NPs size and different concentrations of PAHz as stabilizer to prepare stable o/w PEm system of olive oil (volume fraction, 20%). Therefore in the current study, we have utilized the resulting PAHz–Ag NC stabilized PEm to study influence of NaCl concentration on the o/w PEm stability. Various analytical techniques like optical microscopy, dynamic light scattering (DLS), conductivity, and rheological analysis were used to present emulsion characterization. This study will give further information about influence of NaCl on the PAHz–Ag NC stabilized emulsions at different temperatures for further industrial applications where salt plays important role. We finally report the rheological investigation to study the effect of NaCl on PEm at various temperatures.

## 2. Experimental work

### 2.1. Materials and methods

Tetrahydrofuran anhydrous (THF, Spectrochem, >99.5%), methyl acrylate (SDF Chemicals, >99.0%), tetrabutyl ammonium bromide (TBAB, SDF Chemicals, 99.0%), potassium bromate (SRL, 99.5%), sodium bisulphite extrapure (SDF Chemicals), sodium chloride extrapure (NaCl, SDF Chemicals, 99.5%), hydrazine hydrate (SDF Chemicals, 99.0%), methanol (SDF Chemicals, 99.0%), silver nitrate extrapure (AgNO_3_, SDF Chemicals, 99.8%), an olive oil having density of 0.89 g cm^−3^ at 20 °C (Figaro, B00X7RJPSG) was purchased and utilized for the preparation of o/w PEm. DI water from Millipore® Elix-10 was used for all experiments.

**Fig. 1:**
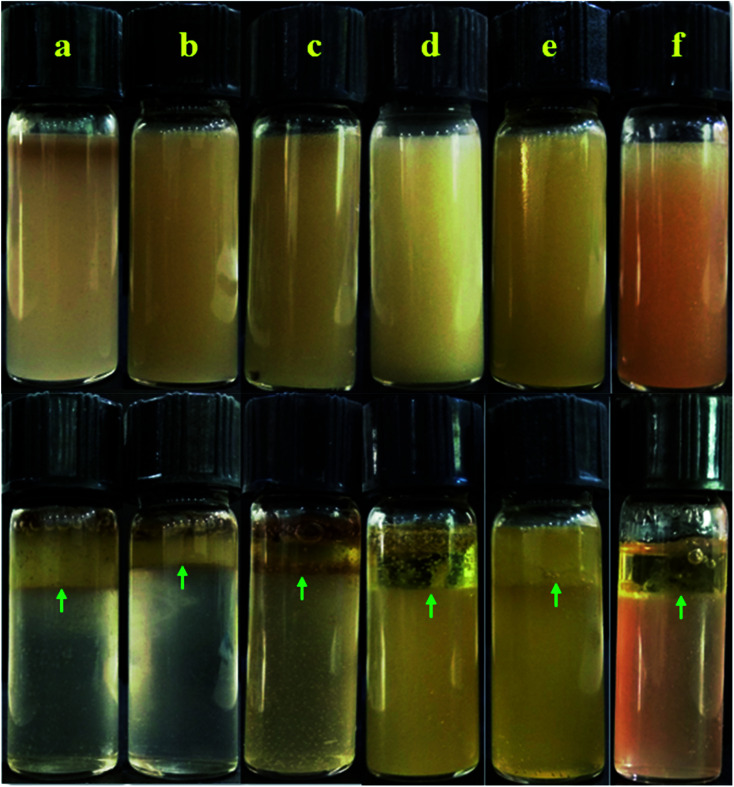
Visual images of (a) PEm-15-1, (b) PEm-15-3, (c) PEm-15-5, (d) PEm-25-1, (e) PEm-25-3, and (f) PEm-25-5 emulsions. Oil separation from emulsion is shown by green arrows in images.

The effect of sodium chloride (NaCl) on PEm stabilized by polyacryloyl hydrazide (PAHz)–Ag NC and olive oil was studied with varying concentrations of polymer and NaCl. The detailed procedure for preparation of PAHz and PAHz–Ag NC, and details of silver NPs size was reported our previous work.^4^ Olive oil and aqueous solution of PAHz–Ag was taken in the ratio of 1 : 4 and mixed for 5 minutes using an industrial mixer, then required amount of NaCl was added to emulsion and further mixed for 5 min to get the final PEm. Equal amount of each emulsion sample was transferred into a separate cleaned vessel immediately after preparation and kept undisturbed to check their time dependent stability. The emulsion was prepared for the different concentrations of PAHz (10, 15, 20, and 25 wt%) and with the varying concentrations of NaCl (0.5, 1.0, 2.0, and 3.0 wt%). The prepared emulsion was creamy in color and started creaming at the upper layer during the course of the time, this can be understood by ocular inspection of emulsions (Fig. 1). The emulsion was characterized by ocular inspection, optical microscope, dynamic light scattering method, electrical conductivity, and rheological study.

### 2.2. Optical microscopic, TGA, and DLS analysis

To visualize emulsion morphology, droplets packing, and size of droplets, a drop of fresh emulsion was placed on a clean glass slide and kept undisturbed to observe under the optical microscope (BA310, Motic®), all the images captured using inbuilt digital camera (Moticam-10) and analyzed using inbuilt software in the microscope.

TGA (thermogravimetric analysis) was conducted for emulsions to understand their thermal stability. It was understood by studying loss of initial mass of emulsion with increasing temperature. All the experiments were conducted using STA 1100 (LINSEIS) from ambient temperature to 500 °C (at a rate of 10 °C min^−1^) under nitrogen atmosphere. ∼15 mg of fresh emulsion sample was used for analysis.

Droplet size distribution and zeta (*ζ*)-potential were measured by Dynamic light scattering (DLS) technique (Zetasizer Nano ZS, Malvern Panalytical®). The instrument was incorporated with Non-Invasive Back Scatter technology (NIBS) to produce highest sensitivity in the results. All the measurements was done at 25 °C and the reproducibility of results was checked by repeating at least thrice and uncertainty in results was found up to ±3.0%.

### 2.3. Electrical conductivity measurements

Electrical conductivity of emulsion can be changed by the addition of salt and with varying temperature.^38,39^ The electrical conductivity of o/w PEm was determined by conductivity meter (Model: HI98129, Hanna Instruments®). Conductivity meter was calibrated with KCl solution and electrode was properly cleaned and dried before conducting each measurement and all the conductivity measurements was done at room temperature. The electrode of conductivity meter was dipped in o/w PEm sample and wait until readings get stable. The measurements were repeated thrice to get reproducibility and significant difference was not found.

### 2.4. Rheological characterization

A compact rheometer (MCR-52 Anton Paar®) was used to carry out rheological measurements of all emulsion samples. Fresh emulsion was used for conducting both shear and dynamic modes to minimize coalescence of oil droplets and oil leakage from emulsion. The bob and cup assembly was used for all measurements, the same assembly was used in our previous study.^40^ Before conducting measurement bob and cup assembly was properly cleaned and dried. Approximately 6 mL of fresh emulsion was carefully taken into a bob and cup assembly, then it was closed tightly and inserted into rheometer chamber where it was maintained by set temperature. Before starting each measurement sufficient time was given to the sample to get uniform temperature. Shear analysis was done for a wide range of shear rate from 1 to 3000 s^−1^ and frequency sweep analysis was in the range of 1 to 100 rad s^−1^ of angular frequency at strain amplitude 5%. All these measurements were done by Rheoplus software which was in-built interface of rheometer. After successful completion of a measurement, emulsion sample was discarded and bob and cup assembly was properly cleaned with toluene and water then it was rinsed with deionized water, later it was dried before conducting next measurement. The temperature inside the rheometer was controlled by external temperature controller (ESCY IC201, −10 to 130 °C). All the measurements were repeated thrice to check their reproducibility and uncertainty in the results were found in between ±0.6–12.0%.

## 3. Results and discussions

A set of o/w PEm were prepared in presence of a predetermined concentration NaCl in the aqueous solution. The emulsion compositions were studied with the help of microscope, DLS, electrical conductivity, and thermogravimetric analysis. Next, rheological properties (shear and oscillatory modes) of PEm with NaCl are presented with suitable comparison.

### 3.1. Characterization of o/w PEm

#### 3.1.1. Visual appearance and microscopic characterization of PEm

**Fig. 2:**
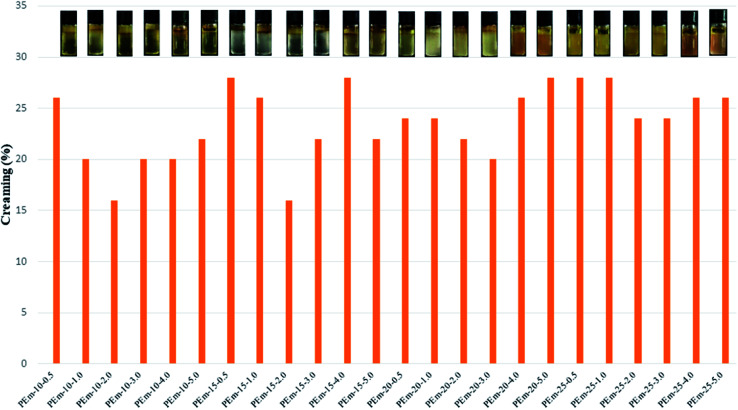
Percentage of creaming in different emulsions after storage period of one week. Here, % creaming = (HC/HT) × 100, HC = height of cream, HT = total height of emulsion in vessel.

The nomenclature (used in this study) and compositional details of PEm is provided in Table 1. PAH–Ag NPs of varying concentration *viz.*, 10, 15, 20, and 25 wt% were used for PEm preparation. NaCl amount was varied from 0.5 to 5.0 wt% as shown in Table 1. PAHz–Ag NPs, consisting of 5–25 wt% PAHz and silver (Ag) NPs of *D*_avg_ 23–10 nm, were synthesized using method described in our previous work.^4^ Olive oil was used to prepare all o/w emulsion samples. The density of olive oil was lighter (0.89 g cm^−3^ at 20 °C) than the aqueous PAHz phase and therefore, oil droplets moved upward during the course of time (creaming phenomenon). Therefore, after preparation, all emulsion samples were kept undisturbed and check for their stability; fresh emulsion was creamy/light yellow in color and there was no phase separation as shown in Fig. 1. During the course of time, the top layer of emulsion progressively turned to thick due to creaming and lower layer was still in emulsion, which indicates that some emulsion droplets moved upward (Fig. 1). The upper layer (cream) of emulsion was different for different NaCl concentrations. It was observed that creaming for all emulsions decreased with increasing NaCl concentration from 0.5 to 3.0 wt%, and afterwards it increased for NaCl concentration of 4.0 and 5.0 wt% as shown in Fig. 2. For high NaCl concentration, most of the oil droplets were in top layer of emulsion as envisaged numerically by percentage of creaming in Fig. 2. This indicates that NaCl concentration plays important role in the stability of PAHz–Ag stabilized PEm. Thus, emulsions stabilized by 10 wt% PAHz (lower polymer concentration) were markedly influenced by the addition of NaCl and therefore, the emulsions (PEm-10-4 and PEm-10-5, Table 1) showed least stability of one day. It has been found that increasing concentration of PAHz increases the stability of PEm^4^ and therefore, emulsions of 25 wt% PAHz–Ag composition (PEm-25-0.5 to PEm-25-5) showed least creaming (Table 1) and highest creaming stability of 7 days was measured for PEm-25-2 and PEm-25-3, consisting of 2 and 3 wt% NaCl, respectively (Table 1). Thus, it can be concluded that salt tolerance capacity of PEm can be improved by increasing polymer concentration in system, which is of key importance for emulsion usage at complex saline conditions.

**Table 1:** Composition, nomenclature, stability, and electrical conductivity details of PEm emulsions

Composition details	PAHz concentration (wt%)	NaCl (wt%)	Nomenclature	Stability (days)	Electrical conductivity (μS)
500 μL of AgNO_3_ (20 mM L^−1^) in 50 mL of PAHz aqueous solution PAHz (0.1 to 0.25 g mL^−1^)	10	0.5	PEm-10-0.5	02	396
		1.0	PEm-10-1	02	842
		2.0	PEm-10-2	02	14 110
		3.0	PEm-10-3	02	15 640
		4.0	PEm-10-4	01	17 250
		5.0	PEm-10-5	01	18 830
	15	0.5	PEm-15-0.5	02	1944
		1.0	PEm-15-1	02	13 220
		2.0	PEm-15-2	03	15 640
		3.0	PEm-15-3	03	48 100
		4.0	PEm-15-4	03	62 130
		5.0	PEm-15-5	02	71 610
	20	0.5	PEm-20-0.5	04	13 730
		1.0	PEm-20-1	04	17 250
		2.0	PEm-20-2	05	39 000
		3.0	PEm-20-3	06	68 800
		4.0	PEm-20-4	03	75 240
		5.0	PEm-20-5	03	79 620
	25	0.5	PEm-25-0.5	06	10 000
		1.0	PEm-25-1	06	15 740
		2.0	PEm-25-2	07	25 100
		3.0	PEm-25-3	07	27 000
		4.0	PEm-25-4	05	32 200
		5.0	PEm-25-5	04	34 610

**Fig. 3:**
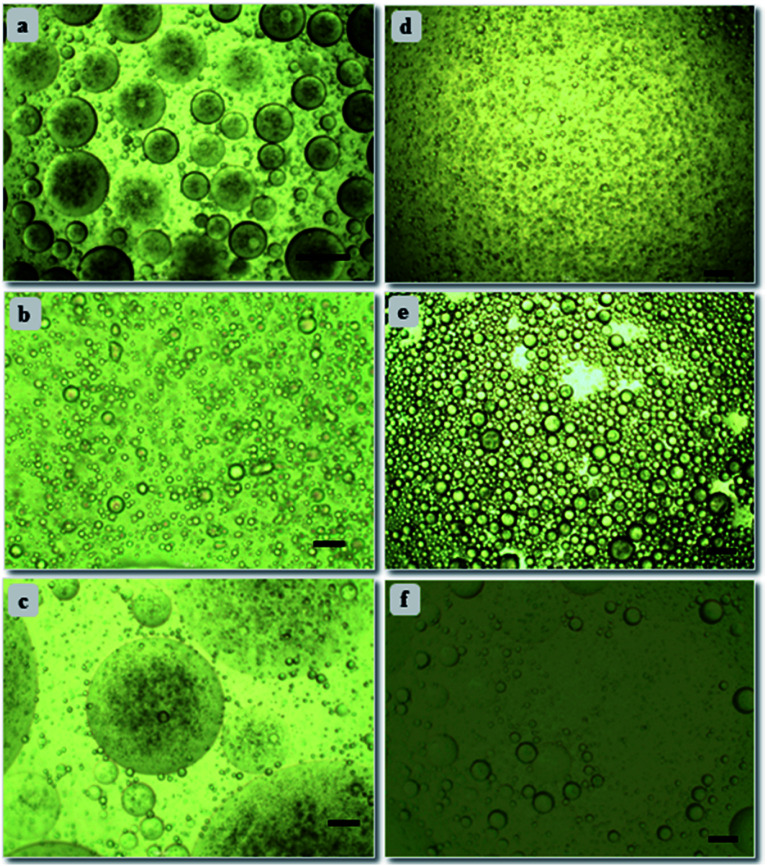
Optical microscopic images of (a) PEm-15-1, (b) PEm-15-3, (c) PEm-15-5, (d) PEm-25-1, (e) PEm-25-3, and (f) PEm-25-5 emulsions at ambient conditions. Scale bar = 30 μm. Refer Table 1 for nomenclature.

**Fig. 4:**
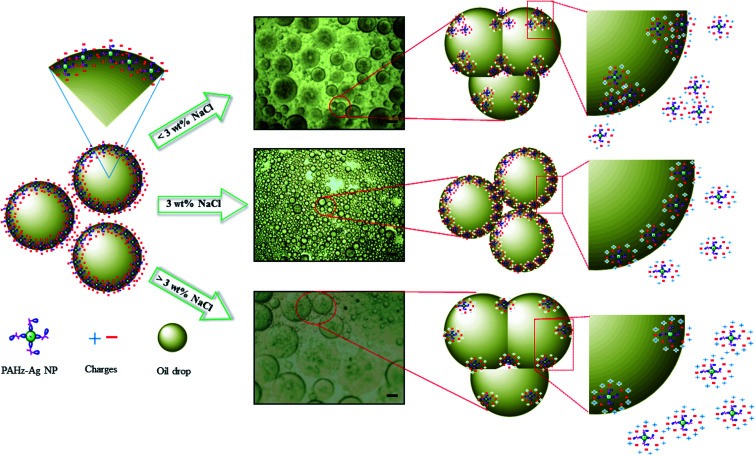
Proposed mechanism for the impact of NaCl on droplet stabilization of Pickering emulsion stabilized by PAHz–Ag nanocomposites. Scale bar in microscopic images of emulsion is 30 μm.

Further, emulsion stability in context of droplet size and their arrangement can be analyzed through optical microscope.^41,42^ Microscopic study was conducted for all concentrations of emulsion samples; emulsion stabilized by 10 wt% PAHz–Ag composition with 0.5 wt% NaCl (PEm-10-0.5) (see Table 1) exhibited significant number of droplets of large size. With increasing NaCl concentration (0.5 to 2.0 wt%), the droplets exhibited slight decrease in size, however, droplet packing did not change much. On the other hand, with further increase in NaCl concentration (3 to 5 wt%, PEm-10-4/5), droplets exhibited significant increase in size and moreover, droplet packing almost disappeared. For PEm-10-5 (10 wt% PAHz–Ag and 5 wt% NaCl), the droplets are significantly bigger in size which indicates that emulsion exhibited droplet coalescence once NaCl concentration in system becomes greater than 3 wt%. Microscopic studies were also performed for other emulsion compositions of 15, 20, and 25 wt% PAHz, where NaCl concentration around 3 wt% was found to be the optimum salinity in emulsion system (Fig. 3). Without NaCl, the electrostatic repulsion between dispersed nanocomposites remained undisturbed by the presence of other ions and therefore, the droplet surface was covered by non-aggregated nanocomposites as depicted in Fig. 4. The scheme of stabilization and destabilization in PEm as a function of NaCl concentration is shown through proposed mechanism in Fig. 4. NaCl inclusion disturbed the state of electrisation repulsion in the system resulting some nanocomposites exhibited attraction which led to aggregation.^43,44^ Since the concentration of NaCl was low (<3 wt%), it could not affect the entire population of nanocomposites resulting aggregation dominated between only some nanocomposites. It reduces the overall surface coverage of nanocomposites as a result, droplets partially exhibited coalescence. This might be the reason of finding large size droplets at low NaCl concentration in microscopic image in Fig. 3a. However, increasing NaCl concentration progressively revived the state of electrostatic repulsion which unfolded the aggregated nanocomposites, indicating NaCl concentration around 3 wt% was the inversion point at which emulsion exhibited superior stability, enhanced interfacial adsorption, and minimal droplet coalescence. This is in agreement with the research findings of Xu *et al.*^34^ who confirmed that concentration of salt plays vital role in the stability of emulsion by controlling coalescence of droplets. Further increase (>3 wt%) probably dominated the ionic strength of solution by NaCl ions which eventually salted-out the electrostatic repulsion of nanocomposites and these nanocomposites re-dispersed back in the suspension (Fig. 4), consistent with the findings in literature.^34^ However, the effect of salinity on emulsion properties was lower for 25 wt% PAHz–Ag composition, which can envisaged through minimal droplet coalescence in Fig. 3d–f. The credit may be given to high concentration of PAHz–Ag NP system and salt ions could not screen the entire population of NPs resulting this emulsion exhibited minimum destabilization at high saline environment (concentration >3 wt%). Thus, from microscopic study, it can be concluded that the salt concentration around 3.0 wt% is the inversion point from emulsion to become stable ↔ unstable.

#### 3.1.2. DLS and electrical conductivity measurements

**Fig. 5:**
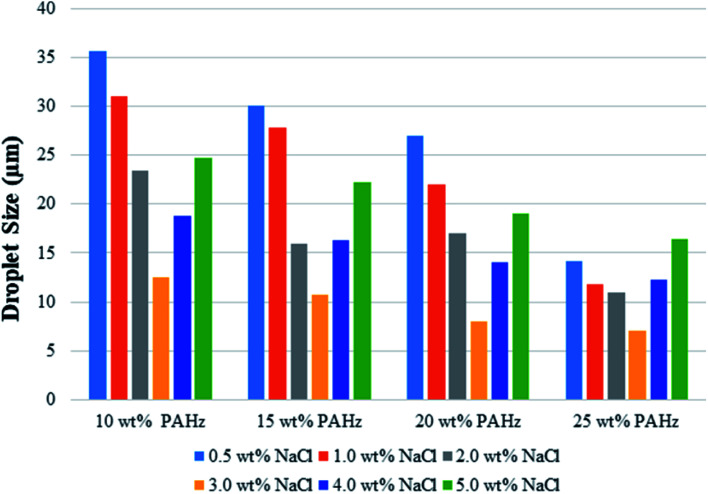
DLS based average droplet size of Pickering emulsions stabilized by 10, 15, 20, and 25 wt% PAHz–Ag nanocomposites in presence of different ionic strength (0.5–5 wt% NaCl).

**Fig. 6:**
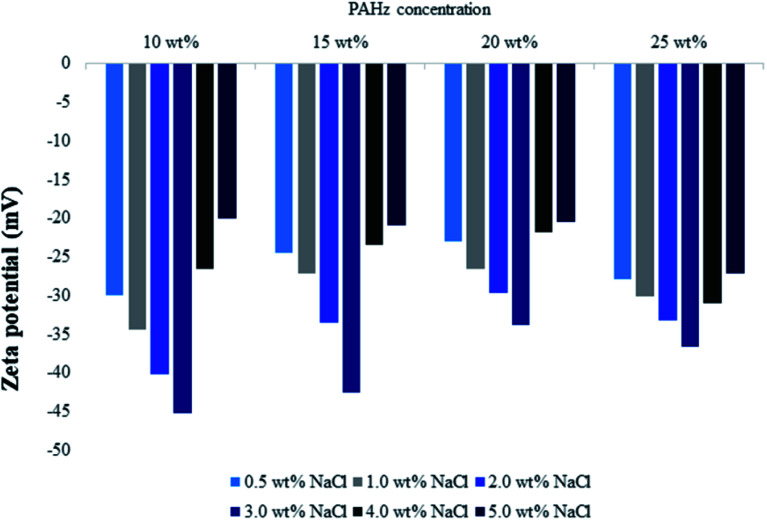
Zeta potential measurements of Pickering emulsions stabilized by 10, 15, 20, and 25 wt% PAHz–Ag nanocomposites as a function of different ionic strength (0.5–5 wt% NaCl).

DLS measurements were conducted for all o/w emulsion samples to further see their size-distribution and zeta-potential. All the samples used for DLS study were fresh and diluted 50 times before analysis. DLS gives the average size distribution of colloidal suspensions and the results were shown in Fig. 5. Average size of sample PEm-10-0.5 was found to be 35.6 μm and for PEm-10-3, it was 12.52 μm (Fig. 5). The size decreased by approximately 66% with increase in NaCl concentration from 0.5 to 3.0 wt% which confirmed enhanced NP adsorption on o/w interface. However, further increase in NaCl concentration showed reverse behavior and droplet size increased by 50% (24.66 μm, Fig. 5) for PEm-10-5 (for 5.0 wt% NaCl), which indicates that NaCl concentration >3 wt% promoted droplet coalescence in emulsion. Similar results were observed for 15, 20, and 25 wt% PAHz–Ag compositions in presence of NaCl (see Fig. 5). O/w emulsion stabilized by 25 wt% PAHz–Ag with 0.5 wt% NaCl (PEm-25-0.5) exhibited average droplet size of 14.15 μm which decreased by 50% at 3.0 wt% NaCl concentration (7.02 μm for PEm-25-3). With further increase in NaCl concentration, droplet size increased to 16.36 μm for PEm-25-5 (see Fig. 5), consistent with microscopic results. Zeta-potential measurements were performed to envisage changes in stability as colloidal suspension exhibiting zeta-potential lesser than ±30 mV is regarded as unstable.^45^ Zeta-potential of emulsion samples was greater than −30 mV (Fig. 6), which confirms that all emulsions were stable suspensions. In addition, emulsion till 3.0 wt% NaCl showed significant increase in zeta-potential resulting its value increased to −30, −34, −40, and −45 mV for PEm-10-0.5, PEm-10-1, PEm-10-2, and PEm-10-3, respectively (see Fig. 6). Zeta-potential results for other compositions exhibited similar trends as shown in Fig. 6. These findings are in accordance with microscopic and size distribution results where NaCl concentration ≤3 wt% showed enhanced emulsion stability with least droplet coalescence. For NaCl concentration >3 wt%, zeta-potential showed reverse trend as its value decreased as shown in Fig. 6. Minimum value of zeta potential was −20, −21, −20.5, and −27, for PEm-10-5, PEm-15-5, PEm-20-5, and PEm-25-5, respectively for 5 wt% NaCl, which are lower than the permissible limit (−30 mV) of stable colloidal suspension.

The electrical conductivity of emulsions can change due to change in their composition and concentration of aqueous phase, and also due to change in aqueous phase salinity.^46–48^ Thus, it is pertinent to measure electrical conductivity of PEm in presence of varying salinity of aqueous phase of PAHz–Ag NPs. NaCl dissolution in aqueous phase (aqueous PAHz–Ag) increases the electrical conductivity o/w emulsion.^49^ Electrical conductivity measurements are presented for two factors which can affect the electrical conductivity of emulsions; change in NaCl concentration and change in aqueous phase concentration. Emulsion PEm-10-0.5 showed electrical conductivity of 396 μS (see Table 1). Increase in conductivity was observed with the increase in NaCl concentration; at 2 wt% NaCl, PEm-10-2 conductivity was determined as 14 110 μS whereas at high NaCl concentration (4 and 5 wt%), increase in conductivity was less (Table 1). It is also well established that increasing concentration of aqueous phase increases the conductivity^39,49^ and therefore, the increased conductivity of PEm-20-0.5 (20 wt% PAHz–Ag + 0.5 wt% NaCl, 13 730 μS) was the result of increase in PAHz concentration. Thus, both PAHz and NaCl collectively affected the conductivity of emulsion. It is to be noted here that change in conductivity with increasing NaCl was comparatively less for high PAHz concentration (25 wt%); 10 000 μS for PEm-25-0.5 (at 0.5 wt% NaCl) increased to only 34 610 μS for PEm-25-5 (at 5 wt% NaCl). This might be due to high concentration of PAHz that significantly restricted the upward movement of emulsion droplets (under gravitational action) and emulsion thus exhibited more non-conductive oil phase in suspension during conductivity measurements. With more oil phase, conductivity did not increase much. The results suggest that at low and high NaCl concentration, emulsion samples did not exhibit greater stability for all PAHz concentrations. However, 25 wt% PAHz–Ag composition treated with NaCl ∼ 3 wt% is remarkable for emulsion applicability in saline environment where conventional emulsions show challenges.

#### 3.1.3. Thermal stability of PEm

**Fig. 7:**
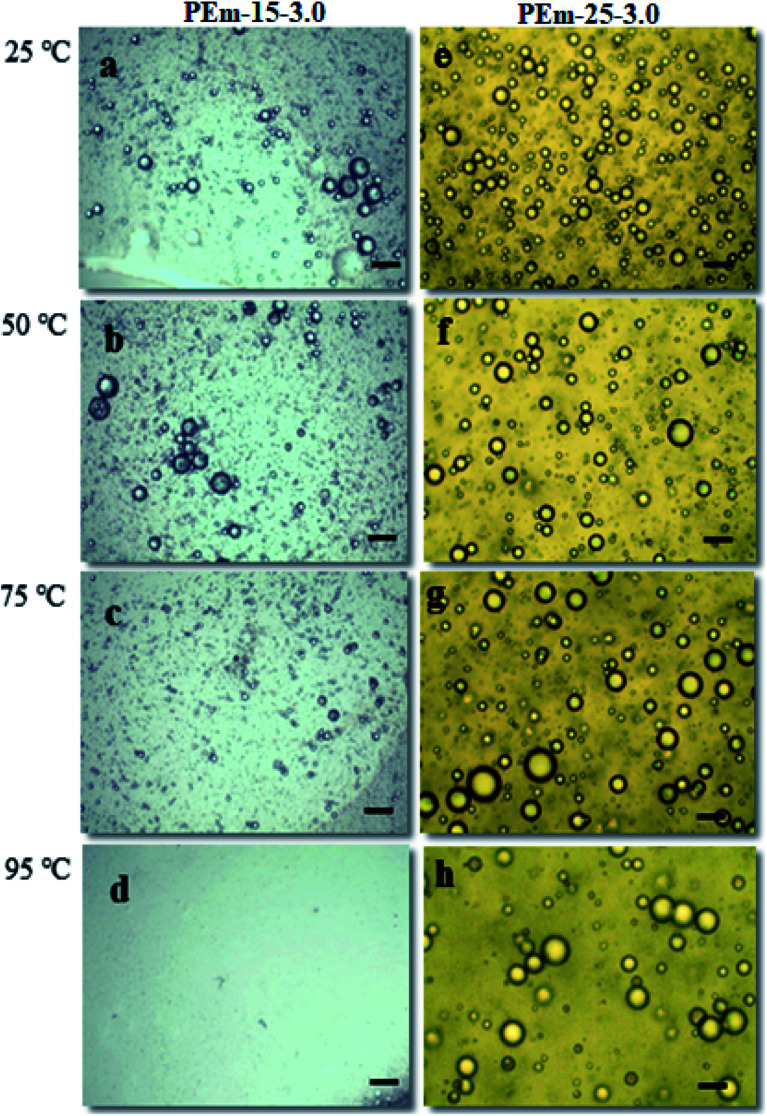
Effect of increasing temperature on droplet stability of PEm-15-3 [(a) 25; (b) 50; (c) 75; (d) 95 °C] and PEm-25-3 [(e) 25; (f) 50; (g) 75; (h) 95 °C] emulsions.

The effect of temperature on emulsion stability was studied using a microscope equipped with thermal stage. An emulsion sample was taken on microscopic slide and temperature is progressively increased *viz.*, 25, 50, 75, and 95 °C. Emulsions stabilized by 15 and 25 wt% PAHz–Ag NPs with salt (NaCl = 3 wt%) were investigated for thermal stability and the results are shown in Fig. 7. It was observed that Brownian movement of emulsion droplets increased with increasing temperature resulting smaller droplets coalesced and converted into bigger droplets. Thus, temperature reduced the stability of emulsion as droplets progressively disappeared with increasing temperature (Fig. 7). Typically, when temperature increases either emulsion droplets coalesce or forms bigger droplets.^50^ In addition, it is also established that increasing temperature leads to rupturing of interfacial film of droplets consequently, smaller droplets disappear from the emulsion.^50^ From Fig. 7a–c, it is evident that droplets were seen with proper packing up to 75 °C of 15 wt% PAHz–Ag. Since most of the emulsion droplets cream at 95 °C of 15 wt% PAHz–Ag, the emulsion body hardly consisted of no droplets to be seen in Fig. 7d. However, emulsion stabilized by 25 wt% PAHz–Ag NC (PEm-25-3) showed droplets with proper packing (see Fig. 7e–h). In addition, thermal stability of 25 wt% PAHz–Ag emulsion (PEm-25-3) was higher due to least effect of increasing temperature on droplets.

**Fig. 8:**
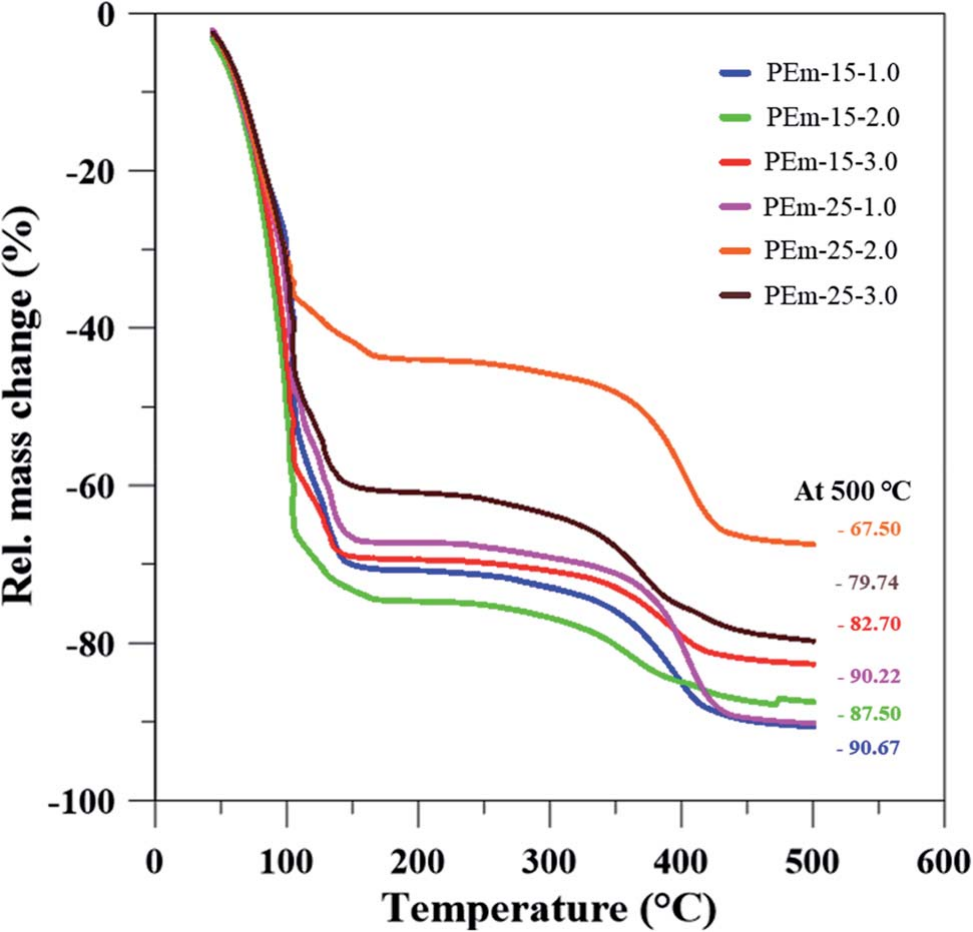
Thermal stability (TGA) analysis for mass loss determination in emulsions stabilized by 15 and 25 wt% PAHz–Ag NC in presence of different NaCl concentration *viz.*, 1, 3, and 5 wt%.

To further understand thermal stability of emulsions, TGA analysis was conducted for same emulsions stabilized by 15 and 25 wt% PAHz–Ag NPs with NaCl (1, 3, and 5 wt%). TGA analysis provides information about emulsion thermal stability and its solid content at a particular temperature.^40,51^ From Fig. 8, it is clear that initial loss in mass of emulsion was due to the loss of water content. In addition, it can be observed that addition of NaCl elevated thermal stability of emulsion. As a result, ∼87% mass loss of emulsion PEm-15-3 (composition 15 wt% PAHz–Ag + 3 wt% NaCl) significantly reduced to ∼67% mass loss for emulsion PEm-25-3 (composition 25 wt% PAHz–Ag + 3.0 wt% NaCl) as shown in Fig. 8.

### 3.2. Rheological characterization

#### 3.2.1. Shear rheological properties

**Fig. 9:**
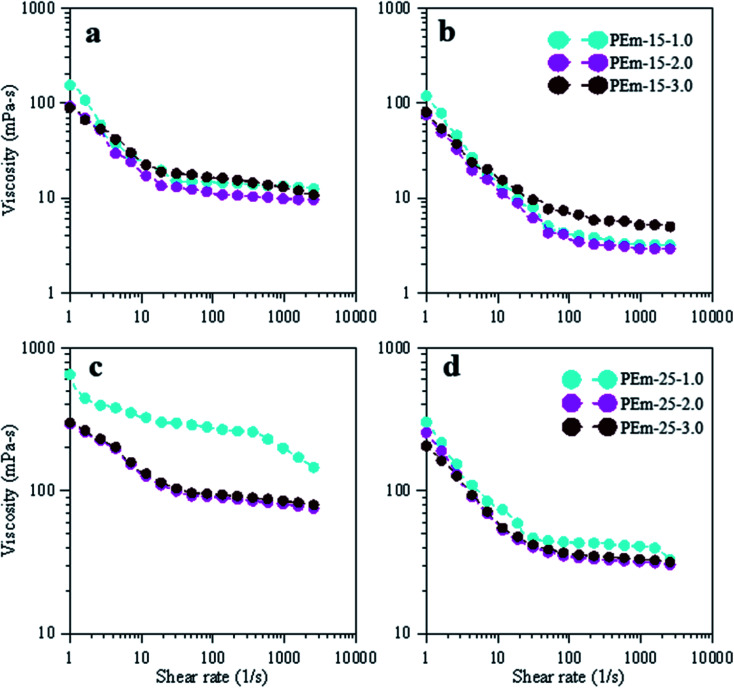
Effect of NaCl (1, 3, and 5 wt%) on viscosity and shear thinning profiles of emulsions, stabilized by 15 and 25 wt% PAHz–Ag NC, at 25 °C (a and c) and 95 °C (b and d).

Flow properties are vital among the physical properties of emulsions and rheological investigation is a best method to understand the influence of electrolytes on these properties of emulsions.^31,52–54^ Factors such as effect of salts, concentration of aqueous phase, and temperature can lead to significant change in the viscosity of an emulsion.^4,28,35,55^ This section presents shear rheological properties of emulsions stabilized by low (15 wt%) and high (25 wt%) compositions of PAHz–Ag NC, and the impact of various ionic strength (1.0–5.0 wt%) on these properties is discussed. Fig. 9 shows viscosity data of PEm as a function of NaCl concentration and temperature. It was observed that all emulsion samples showed non-Newtonian shear thinning behavior with increasing shear rate; viscosity decreases with shear rate. Initial viscosity of PEm-15-1 at 25 °C was 154.7 mPa s (at shear rate of 1 s^−1^) and with increasing shear rate, this viscosity was measured to be 37.5 mPa s (at shear rate of 4.35 s^−1^) and 12.4 mPa s (at a shear rate of 3000 s^−1^) as shown in Fig. 9a. The decrease in emulsion viscosity is attributed to droplet deflocculation against shear deformation.^52^ With increasing NaCl concentration, the viscosity of emulsion further decreased and at 3 wt% NaCl (PEm-15-3), the initial viscosity was measured as 92.49 mPa s (a decrease of 40%). This reduction in emulsion viscosity is expected as NaCl relaxed the packing of droplets and made less concentrated.^37^ Emulsion viscosity was found minimum (89 mPa s) at 5 wt% NaCl for PEm-15-5 (Fig. 9a). Since emulsion at 5 wt% NaCl was found unstable, most of the droplets moved to the surface and viscosity measured for emulsion represented more or less viscosity of aqueous phase. Similar viscosity trends were observed for other emulsion compositions (10 and 20 wt%) of PAHz–Ag NPs, hence their viscosity data is not shown for brevity.

To understand the effect of temperature, viscosity measurements were conducted on fresh emulsions to avoid the possibility of creaming and phase separation. The emulsion viscosity was measured at different temperature such as 50, 75, and 95 °C. It was found that the viscosity gradually decreased with increase in temperature. However, temperature effect was more on emulsion treated with lower salt concentration resulting initial viscosity decreased by ∼23% for PEm-15-1 at 95 °C (118.83 mPa s) (Fig. 9b) while viscosity of PEm-15-3 decreased by ∼20% (75.25 mPa s at 95 °C) (Fig. 9b). This suggests that PEm-15-3 was relatively more thermally stable than PEm-15-1. The reason is credited to optimum concentration of NaCl (3 wt%) at which NPs provided significant steric barrier against thermal degradation of droplets.^50^ PAHz concentration was increased to 25 wt%, and the viscosity results are obtained to compare the effect of increasing NP concentration on rheological response of emulsion. It was observed that viscosity increased with increasing PAHz concentration as shown in Fig. 9c and d. The initial viscosity of emulsion PEm-25-1 was 652.8 mPa s at 25 °C (Fig. 9c) (viscosity increased by 4X when compared to PEm-15-1). This viscosity decreased with increasing NaCl concentration and its value reached to 294 mPa s at 3 wt% NaCl, which is consistent with the findings of NaCl effect on emulsion viscosity.^37^ It is to be noted here that viscosity of this emulsion at low NaCl concentration decreased more with increasing temperature as evident from Fig. 9d. Since this emulsion (PEm-25-1) exhibited lower thermal stability at low NaCl concentration, the viscosity of emulsion decreased more with increasing temperature. On the other hand, the viscosity of emulsion PEm-25-3 did not decrease much with increasing temperature and emulsion showed stable rheological response at each temperature as shown in Fig. 9d. Thus, PEm-25-3 viscosity was least affected by the change in temperature than the one of PEm-15-3; PEm-25-3 exhibited viscosity decrease of 15% at 95 °C, lesser than 20% decrease of PEm-15-3. Therefore, emulsion stabilized by 25 wt% PAHz–Ag NPs and 3.0 wt% NaCl can be used for high temperature applications.

**Fig. 10:**
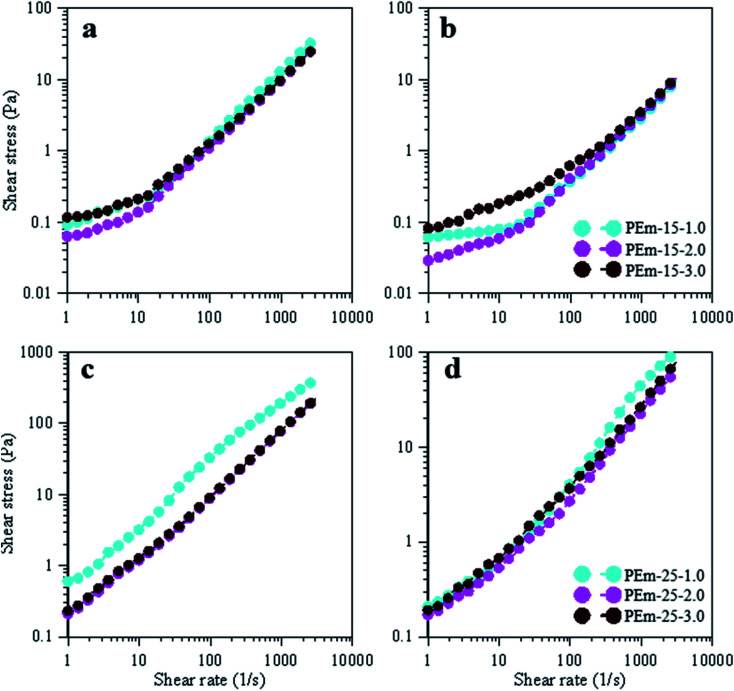
Effect of NaCl (1, 3, and 5 wt%) on yield stress data of emulsions, stabilized by 15 and 25 wt% PAHz–Ag NC, at 25 °C (a and c) and 95 °C (b and d).

Yield stress is another important flow property that provides information on amount of torque required to permanently deform droplets in a suspension.^40,56,57^ Yield stress data in form of shear stress *vs.* shear rate for emulsions (15 and 25 wt% PAHz–Ag stabilized) is shown in Fig. 10. Yield stress for PEm-15-1 was determined as 0.09 Pa (at shear rate 1 s^−1^) at 25 °C. In addition, yield stress decreased with increasing NaCl concentration. At 25 °C, yield stress value of 0.06 Pa was determined for PEm-15-3 (see Fig. 10a). It indicates that the required torque to deform emulsion droplets decreased with increasing NaCl concentration from 1.0 to 3.0 wt%, which suggests that droplet packing at low NaCl concentration was more compacted that the one at 3 wt% NaCl. Therefore, the requirement of initial torque (yield stress) to deform droplet structure at 1.0 wt% NaCl was found higher. This effect of NaCl on yield stress of PAHz–Ag stabilized emulsion is in agreement with the findings reported in literature for PEm.^37^ Further increase in NaCl concentration (PEm-15-5) has slightly increased the yield stress value to 0.11 Pa (Fig. 10a). Yield stress values of 25 wt% PAHz–Ag stabilized emulsion were higher than the ones of 15 wt% PAHz–Ag stabilized emulsions, in line with viscosity results of these emulsions (Fig. 10). PEm-25-1 exhibited yield stress value of 0.60 Pa, which decreased to 0.21 Pa for PEm-25-3 at 25 °C, indication more relaxed droplet structure in PEm-25-3. Yield stress value increased to 0.24 Pa for PEm-25-5. Thus, yield stress results also confirm that NaCl concentration of 3 wt% is favorable for flow properties of PAHz–Ag stabilized emulsions.

Temperature effect on yield stress was also studied for understanding the behavior of emulsions at various temperatures *viz.*, 50, 75, 95 °C (Fig. 10b and d) (data for 50 and 75 °C is not shown). The required torque to deform the emulsion droplets decreased with increasing temperature and for PEm-15-1 (15 wt% PAHz–Ag, 0.5 wt% NaCl), its value was determined as 0.06 Pa (at shear rate 1 s^−1^, 95 °C) (Fig. 10b), which was 33% lesser than yield stress of PEm-15-1 at 25 °C (Fig. 10a). Which suggests at 95 °C droplets were less compacted compared to 25 °C. As the NaCl concentration increased, required torque showed decreasing trend up to 3.0 wt% NaCl (0.02 Pa at shear rate 1 s^−1^ for PEm-15-3) and above 3.0 wt% NaCl concentration yield stress showed increasing concentration (0.08 Pa at shear rate 1 s^−1^ for PEm-15-5) (Fig. 10b). These results are also followed the same trend and in line with yield stress results at 25 °C. The yield stress values of emulsions stabilized with 25 wt% PAHz–Ag at 95 °C were lower than the yield stress values at 25 °C and followed similar trend. Yield stress value of PEm-25-1 at 95 °C (0.20 Pa, at shear rate 1 s^−1^) was decreased 66% when compared to PEm-25-1 at 25 °C and as NaCl concentration increased to 3.0 wt% yield stress was decreased (Fig. 10d). PEm-25-3 at 95 °C showed 19% lesser yield stress (0.17 Pa, at shear rate 1 s^−1^) compared to yield stress of PEm-25-3 at 25 °C. Above 3.0 wt% NaCl concentration yield stress was increased and these results were in line with the yield stress results of emulsions stabilized with PAHz–Ag at 25 °C (Fig. 10). This indicates the PEm-25-3 was more stable at 95 °C when compared to other emulsion at various NaCl concentrations. Therefore, PEm-25-3 (25 wt% PAHz–Ag, 3.0 wt% NaCl) can be better suitable for high temperature applications.

#### 3.2.2. Dynamic viscoelastic properties

**Fig. 11:**
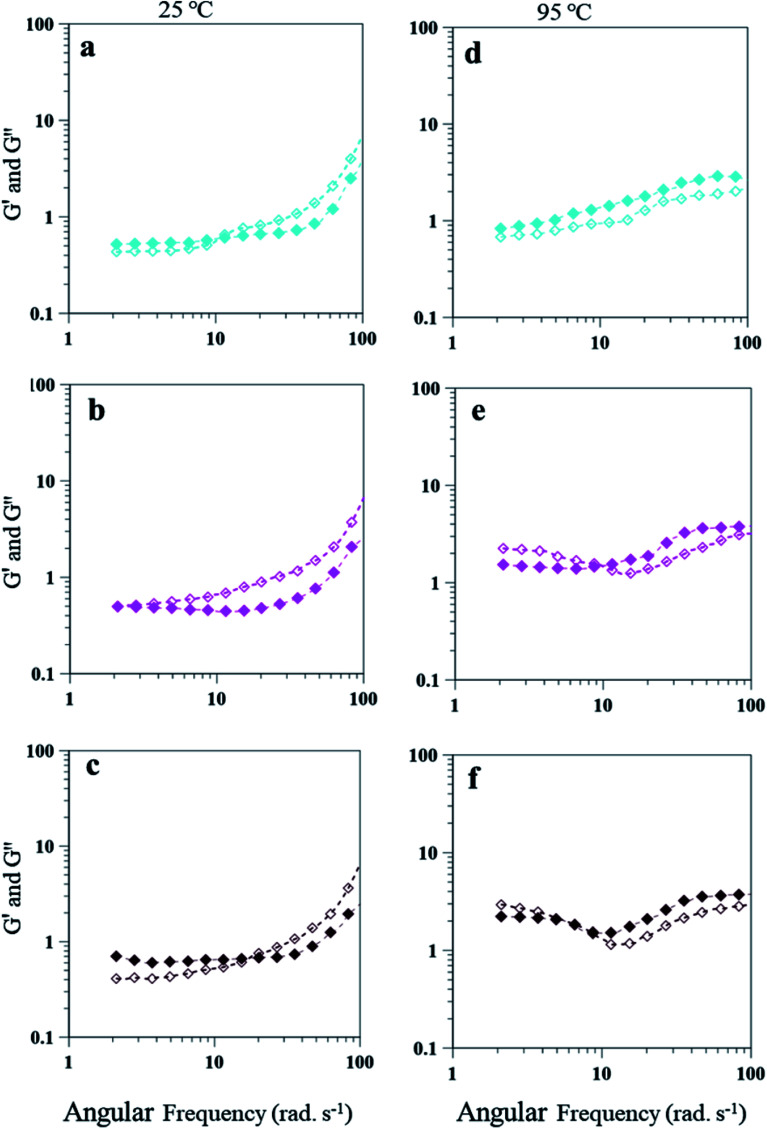
Frequency-sweep measurements of 15 wt% PAHz–Ag NC emulsions as a function of different ionic strength (a) and (d) PEm-15-1 (1 wt% NaCl), (b) and (e) PEm-15-3 (3 wt% NaCl), and (c) and (f) PEm-15-5 (5 wt% NaCl) at 25 and 95 °C, respectively. Filled symbols for *G*′ and empty symbols for *G*′′ are used.

Storage modulus (*G*′) and loss modulus (*G*′′) (Dynamic rheological properties) are useful to understand viscoelastic behavior of PEm.^58,59^ Similar to shear study, emulsions of 15 and 25 wt% PAHz–Ag compositions were studied for dynamic rheology as a function of different NaCl concentration (1.0–5 wt%) and temperature (25 and 95 °C). Frequency sweep analysis was conducted with the angular frequency ranges from 1 to 100 rad s^−1^ at strain amplitude of 5%. Strain amplitude value of 2% was chosen as recommended for dynamic rheology of PEm in literature.^40^*G*′ value of PEm-15-1 was recorded as 0.52 Pa (at 2.12 rad s^−1^, 25 °C), higher than *G*′′ (0.43 Pa) as shown in Fig. 11a. This suggests that PEm-15-1 was elastic in nature but a crossover at 10.5 rad s^−1^ was observed with increasing frequency resulting *G*′′ became higher than *G*′ (Fig. 11a). Since increasing NaCl concentration to 3.0 wt% shifted the crossover at lower angular frequency, emulsion became more viscous like (Fig. 11b). This was attributed to aqueous phase of emulsion at 3.0 wt% NaCl had least interaction with salt and PAHz–Ag NC was properly dispersed between oil and water interface and reduced formation of three dimensional network of NC and enhanced viscous nature of emulsion. *G*′′ and *G*′ values of PEm-15-3 were almost equal (0.5 and 0.49 Pa respectively) and emulsion exhibited viscous like nature over the entire range of frequency explored (Fig. 11b). However, reverse in emulsion behavior was observed once NaCl concentration became more than 3 wt%. For PEm-15-5, crossover occurred at 18.4 rad s^−1^, as a result, emulsions showed elastic nature at lower values of frequency explored (Fig. 11c). This is attributed to droplet flocculation and creaming; at high NaCl concentration, emulsion exhibited flocculated droplet structure which was elastic and compact as evident from higher *G*′ at low frequency levels. With increasing frequency, droplet structure was broken and droplets re-dispersed in emulsion resulting crossover occurred at high angular frequencies. At 95 °C, these emulsions showed reduction in viscous nature as *G*′′ became lower than *G*′ over most of the frequencies explored (Fig. 11d–f). For PEm-15-1, *G*′ (0.83 Pa) dominated *G*′′ (0.68 Pa) without showing any crossover point (Fig. 11d). This is attributed to temperature effect on emulsion viscosity; viscosity of emulsions reduced at high temperature resulting it caused significant decrease in viscous component than elastic component. However, for emulsions *viz.*, PEm-15-3 and PEm-15-5, *G*′′ was found slightly higher than *G*′ with crossover at 10.5 and 5.96 rad s^−1^, respectively. Since these emulsions were more viscous due to increased dissolution of salt ions, *G*′′ slightly increased and remained higher for some frequencies with crossover at 10.5 (PEm-15-3) and 5.96 (PEm-15-5) as shown in Fig. 11e and f.

**Fig. 12:**
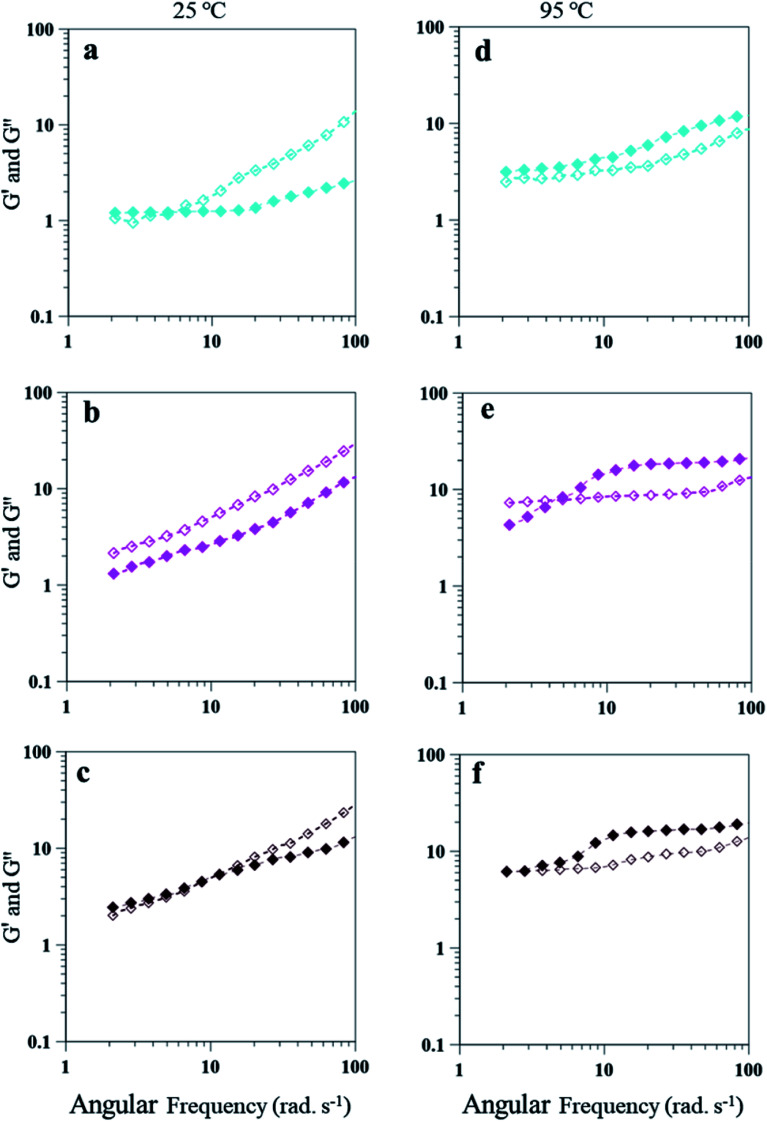
Frequency-sweep measurements of 25 wt% PAHz–Ag NC emulsions as a function of different ionic strength (a) and (d) PEm-25-1 (1 wt% NaCl), (b) and (e) PEm-25-3 (3 wt% NaCl), and (c) and (f) PEm-25-5 (5 wt% NaCl) at 25 and 95 °C, respectively. Filled symbols for *G*′ and empty symbols for *G*′′ are used.

The emulsion composition of 25 wt% PAHz–Ag exhibited higher *G*′ and *G*′′ values due to higher viscosity of these emulsions. Similar to 15 wt% PAHz–Ag, these emulsions also exhibited dominating viscous behavior for all NaCl concentrations at 25 °C. However, for lower NaCl concentration such as 1.0, initially *G*′ dominated the behavior with crossover at 5.43 rad s^−1^ (Fig. 12a). This crossover was observed at lower angular frequencies when compared with the ones of 15 wt% PAHz–Ag emulsion, which indicates that NaCl affected the viscoelastic behavior of these emulsions differently. Therefore, it is very important to investigate the salt role on rheology of PEm. Emulsion behavior at 3 wt% NaCl (PEm-25-3) is *G*′′ dominated over the entire range of angular frequency explored. With increase in NaCl concentration to 5 wt%, emulsion behavior slightly shifted to elastic with *G*′ dominance till 10 rad s^−1^ (see Fig. 12b). This can be possible if the dispersed NP stabilized droplets coalesce and separate from the emulsion, resulting viscous nature of emulsion will decrease as evident from the evolution of elastic nature at 5 wt% NaCl. This suggests that NaCl concentration >3 wt% is detrimental to emulsion stability and its rheological properties. The rheological properties of these PEm is also influenced by high temperature, similar to the results of 15 wt% PAHz–Ag emulsion. At 95 °C, the behavior of emulsion is elastic for all NaCl concentrations which is different than the one at 25 °C (Fig. 12d–f). The results indicate the rheology of PAHz–Ag stabilized emulsion is differently affected by different operating conditions such as saline environment and temperature. Therefore, the rheological properties of these emulsions must be envisaged to broaden their applicability in different applications.

## 4. Conclusion

The study revealed that, PEm based on PAHz–Ag NC can be used under moderate to strongly saline environments without notably compromising on its stability. The amount of PAHz–Ag NC in the PEm is critical to determine the extent of stability of PEm under saline environment. An increase in PAHz–Ag NC from 10 to 25 wt% increased the stability of PEm by 7 times. Emulsion stabilized with lower PAHz–Ag NC (10, 15, 20 wt%) were unstable (enhanced droplet coalescence) in presence of salt concentrations. However, emulsion with 25 wt% PAHz–Ag NC + 3 wt% NaCl (PEm-25-3) showed superior stability with an average droplet size of 7 μm, which was 80% lesser than average droplet size of PEm-10-0.5. The amount of salt in the aqueous solution affected the positioning of PAHz–Ag NC at the interface and its stabilization efficiency. PEm emulsions were also able to handle high temperature conditions up to 95 °C: PEm-25-3 at 95 °C exhibited significant rheological stability with enough droplet packing (least droplet coalescence) and reduced mass loss. Overall, the studied PEm based on PAHz–Ag NC are suitable for use in extreme saline and temperature environments. Further mechanistic investigation is under process to ascertain the role of salt and polymer interaction in these PEms.

## Conflicts of interest

There are no conflicts of interest to declare.

## Supplementary Material
